# Trends in Determinants of Hypercholesterolemia among Chinese Adults between 2002 and 2012: Results from the National Nutrition Survey

**DOI:** 10.3390/nu9030279

**Published:** 2017-03-15

**Authors:** Peng-kun Song, Hong Li, Qing-qing Man, Shan-shan Jia, Li-xiang Li, Jian Zhang

**Affiliations:** 1 National Institute for Nutrition and Health, Chinese Center for Disease Control and Prevention, 27 Nanwei Road, Xicheng District, Beijing 100050, China; spk_8210@163.com (P.-k.S.); manqq@ninh.chinacdc.cn (Q.-q.M.); jssky.good@163.com (S.-s.J.); lilx@ninh.chinacdc.cn (L.-x.L.); 2 Central laboratory of Beijing Center for Disease Control and Prevention, 16 Hepingli Middle Street, Dongcheng District, Beijing 100013, China; benlih@sina.com

**Keywords:** hypercholesterolemia, determinants, socioeconomic characteristics, lifestyle, diet, BMI

## Abstract

Hypercholesterolemia is a known risk factor for cardiovascular diseases and affects a high proportion of the population. This study aimed to assess and compare the determinants of hypercholesterolemia among Chinese adults aged 18 years and above, from 2002 to 2012. The study used a stratified multistage cluster sampling method to select participants. Sociodemographic and lifestyle information was collected during face-to-face interviews. Dietary intake was calculated by 3-day, 24-h dietary records in combination with weighted edible oil and condiments. Hypercholesterolemia was defined as total cholesterol above 6.22 mmol/L (240 mg/dL) from fasting blood samples. The study included 47,701 (mean age 43.0 years) and 39,870 (mean age 51.0 years) participants in 2002 and 2010–2012 surveys respectively. The weighted prevalence of hypercholesterolemia increased from 1.6% (2.1% urban, 1.0% rural) in 2002 to 6.0% (6.4% urban, 5.1% rural) in 2012. The intake of plant-based food decreased but the intake of pork increased over the 10 years. A high intake of protein and pork, alcohol drinking and overweight/obesity were positively associated with hypercholesterolemia. Neither education nor fruit and vegetable intake were associated with hypercholesterolemia. In conclusion, the burden of hypercholesterolemia increased substantially between 2002 and 2012 in China. Unhealthy lifestyle factors and change in traditional dietary pattern were positively associated with hypercholesterolemia. Further research on the role of diet in the development and prevention of hypercholesterolemia is needed.

## 1. Introduction

Abnormal lipid metabolism is an important risk factor for cardiovascular diseases (CVD). It has been shown that one unit increase of logarithmic serum total cholesterol will triple the risk of CVD in men and women [1]. Hypercholesterolemia has been confirmed as an independent risk factor for atherosclerosis and thrombosis, coronary heart disease and ischemic stroke [2,3]. The prevalence of hypercholesterolemia has decreased and mean total cholesterol (TC) levels have fallen by as much as 40 mg/dL in most developed countries during the past several decades because of statin use, healthy diet promotion and national public health policies [4]. However, in China, the mean total cholesterol level is approaching 4.50 mmol/L (173 mg/dL) and the prevalence of hypercholesterolemia increased from 2.9% in year 2002 [5] to 4.9% in 2012 [6]. Although blood lipid levels and the prevalence of dyslipidemia among Chinese residents are still lower than those in Western countries, with the rapid development of the social economy and improvements of living standards as well as the change of lifestyle, the burden of CVD has increased significantly; it can be partly attributed to the increased level of blood lipids.

The associations between sociodemographic factors, diet and lifestyle factors for hypercholesterolemia have been documented in many epidemiological and observational studies [7,8]. Dietary habits and lifestyle among Chinese people have changed a great deal during the last 10 years, especially among middle-aged people. How these changes are linked with cholesterol levels is not well studied in China. There is no study on the association between macronutrients intake and hypercholesterolemia at the national level. Using data from 2002 and 2010–2012 Chinese National Nutrition Survey, the current study aimed to (1) describe the prevalence of hypercholesterolemia; and (2) compare the dietary and lifestyle determinants of hypercholesterolemia among adults aged 18 years and over between 2002 and 2012.

## 2. Materials and Methods

### 2.1. Data Sources

Data used in the study were from the National Health and Family Planning Commission (former Ministry of Health of the People’s Republic of China) Medical Reform Major Program: Chinese Nutrition and Health Surveillance (2010–2012) and the National Nutrition and Health Survey (2002). The two surveys were conducted in 31 provinces, autonomous regions, and municipalities directly under the central government, throughout China (except Taiwan, Hong Kong, and Macao).A stratified multistage cluster sampling method was used to select participants.

In the 2002 survey, the country was divided into 6 strata (big cities, medium and small cities, class 1–4 rural areas) based on geographic characteristics, economy, and social development information provided by the China National Bureau of Statistics and the China Ministry of Health Statistics. The first stage of sampling involved a random selection of 22 districts (urban) or counties (rural) from 6 strata and the second stage involved random selection of three neighborhoods (urban) or townships (rural) from each of the selected districts/counties; and then from each of the neighborhoods or townships, two residential committees (urban) or villages (rural) were randomly selected. Thirty households were randomly sampled from each neighborhood committee or village, and the dietary survey with 3-day 24-h dietary records, combined with food weighing, was conducted.

In the 2010–2012 survey, a multistage stratified with proportional to the population cluster random sampling method was conducted at 150 survey sites of4 types, of which 34 were big cities, 41 were medium and small cities, 45 were general rural areas, and 30 were poor rural areas (according to economy and social development). Random sample selection of 6 neighborhood committees (urban) or 6 villages (rural) conducted equal to the proportion of the local population. Thirty households were randomly sampled from each neighborhood committee or village, and the dietary surveys, with 3-day 24-h dietary records, combining food weighing, were finished. Both surveys were approved by the Ethical Committee of the National Institute for Nutrition and Food Safety of the Chinese Center for Disease Control and Prevention. All the participants provided written informed consent.

A similar survey questionnaire and fasting blood measurements were conducted. A face-to-face interview using a standard questionnaire was conducted at the home of participants and anthropometric measurements were taken at a community health service center, both by trained staff. The questionnaire included general demographic information, such as age, education level, marital status and income, and lifestyle factors, including smoking and alcohol consumption. Weight was measured without shoes and in light clothing to the nearest 0.1 kg. Height was determined to the nearest 0.1 cm without shoes. Body mass index (BMI) was calculated as weight (kg) divided by height (m) squared.

Fasting blood samples were collected by qualified nurses by venipuncture from the antecubital vein before breakfast. The samples were centrifuged at 1500× *g* for 10 min after being left standing for 30 to 60 min. The centrifuged serum samples were transported to a laboratory and stored at −80 °C. The blood collection procedure, processing, and determination were identical in the two surveys. Serum total cholesterol was tested using the cholesterol oxidase method by the Hitachi automatic biochemical analyzer and reagents were from a central laboratory of the Beijing Center for Disease Control and Prevention. According to the Chinese adult dyslipidemia prevention guide (2007 edition), serum total cholesterol concentrations greater than 6.22 mmol/L (240 mg/dL) were considered to be hypercholesterolemia [9].

### 2.2. Inclusion and Exclusion Criteria

In both surveys, participants aged 18 years and above, who were healthy and able to walk freely to the survey location were included in the survey. Those with missing values of basic demographic information, lysed blood samples, were excluded following unified data cleansing rules (samples with missing or illogical data, energy intakes lower than 800 kcal per day; or higher than 5000 kcal per day were excluded).

### 2.3. Statistical Analyses

Data were collected using specialized software, and data analyses were performed using SAS (Statistical Analysis System) for Windows V9.3 (SAS Institute, Cary, NC, USA). National population census data from 2010 were used to calculate weighted serum total cholesterol concentration, prevalence of hypercholesterolemia, dietary intake, and other variables. A sampling weight was assigned to each participant based on the study design. As the age and gender distribution of the Chinese population was different between 2002 and 2010/2012, in the analysis of the prevalence of hypercholesterolemia and mean levels of cholesterol we calculated age and gender standardise values based on the 2010 national census data. China national food composition 2004 [10] and 2009 [11] editions were used to classify food categories and to calculated nutrients intake. PROC SURVEYMEANS and PROC SURVEYFREQ procedures in SAS were used to calculated mean and proportion and their 95% Confident Interval (95% CI).

Three complex sampling logistic regression analyses were used to assess the associations between sociodemographic factors, dietary intake, lifestyle factors and hypercholesterolemia in both surveys. Food/nutrient intakes were categorized as quartiles or tertiles depending on the distribution of consumption. The association between food and nutrient intake and hypercholesterolemia was adjusted for district, age, gender, nationality, education, marital status, income, smoking, drinking, physical activity, BMI and energy intake. A Student *t*-test was used to test the differences in the means of continuous variables, and the Chi-square test was used to test differences in categorical variables. A two tailed *p* value < 0.05 was considered to be statistically significant.

## 3. Results

The demographic characteristics of the participants are shown in Table 1. The final sample sizes were 45,701 in the 2002 survey (15,554 in urban and 30,147 in rural areas) and 39,870 in the 2010–2012 survey (19,841 in urban areas and 20,029 in rural areas).

In the 2002 survey, the participants’ age range was from 18 years to 95 years, and the mean age was 43.0 years (42.7 years urban; 43.4 years rural). The Han population accounted for 91.4% (94.5% of urban and 87.3% of rural areas). Most participants completed junior or senior high school in urban areas, but participants with primary school or below accounted for 47.3% and only 2.6% of participants with college or above in rural areas. The proportion of having a partner was 76.7% in urban and 82.0% in rural areas. Most participants had income below 5000 Yuan RMB (45.1% urban; 85.6% rural).

The mean age was higher in the 2010–2012 survey at 51.0 years (50.5 years urban; 52.3 years rural), which is older than the 2002 survey. Education levels were similar between the two surveys. However, there was a significant increase in the annual income in 2010–2012 survey.

Table 2 shows the age and gender adjusted level of serum total cholesterol (TC) concentration and other lifestyle factors. The mean level of TC increased significantly from 3.93 mmol/Lin 2002 (4.05 mmol/L urban; 3.79 mmol/L rural) to 4.62 mmol/L in 2012 (4.66 mmol/L urban; 4.54 mmol/L rural).Similarly, the overall prevalence of hypercholesterolemia increased significantly from 1.6% in 2002 to 6.0%in 2012 (*p* < 0.001). In men, the prevalence of hypercholesterolemia increased from 1.3% in 2002 to 4.7% in 2010–2012. In women, the increase was even greater (2.0% in 2002, 7.0% in 2010–2012).The increase of the prevalence of hypercholesterolemia was higher among those aged above 55 years as compared with the younger age groups.

The proportion of smokers was 26.3% in urban and 30.0% in rural in the 2002 survey, while, in 2012, the proportion decreased to 23.1% in urban and increased to 30.7% in rural, the total smoking prevalence significantly decreased from 27.9% in 2002 to 25.5% in 2012 (*p* < 0.001). The proportion of drinkers increased significantly from 23.9% (24.6% urban; 23.0% rural) in 2002 to 33.6% (33.7% urban; 33.5% rural) in 2012 (*p* < 0.001). However, the proportion people who exercised more than 150 min per week was not significantly different between two surveys (*p* = 0.195). Mean BMI also showed significantly increasing trends, from 23.1 kg/m^2^ in 2002 to 23.8 kg/m^2^ in 2012 (*p* < 0.001). The proportions of low-weight (<18.5 kg/m^2^), normal weight (18.5–23.9 kg/m^2^), overweight (24.0–27.9 kg/m^2^), and obesity (≥28.0 kg/m^2^) were significantly different between the two surveys (*p* < 0.001).

Weighted food consumption was shown in Table 3, and was classified by different districts and gender after adjusted by age. The mean dietary consumption of rice, wheat, other cereals, vegetables, eggs, fish, milk, red meat offal, other red meat, legumes, tubers, sugar, salt and animal oil decreased significantly from 2002 to 2012 (*p* < 0.05). Pork, cakes and vegetable oil intake increased significantly during the same period (*p* < 0.001) in both genders. No statistical difference was observed in fruits (*p* = 0.114), poultry (*p* = 0.062) and nut intake (*p* = 0.442) among females from 2002 to 2012. In both genders, rice consumption decreased more than 70 g/day.

Table 4 shows the intake of weighted energy and macronutrients in both surveys. Total energy intake decreased more than 200 kcal/day between 2002 and 2012 in both gender. The decrease in energy intake was mainly due to the reduction of carbohydrate intake. However, the percent of energy from fat increased significantly from 32.0% in 2002 to 34.4% in 2012 in men and from 32.2% in 2002 to 34.5% in 2012 in women (*p* < 0.001). In contrast, percentage of energy from carbohydrate and protein decreased from 2002 to 2012 in both genders (*p* < 0.001).

**Figure 1:**
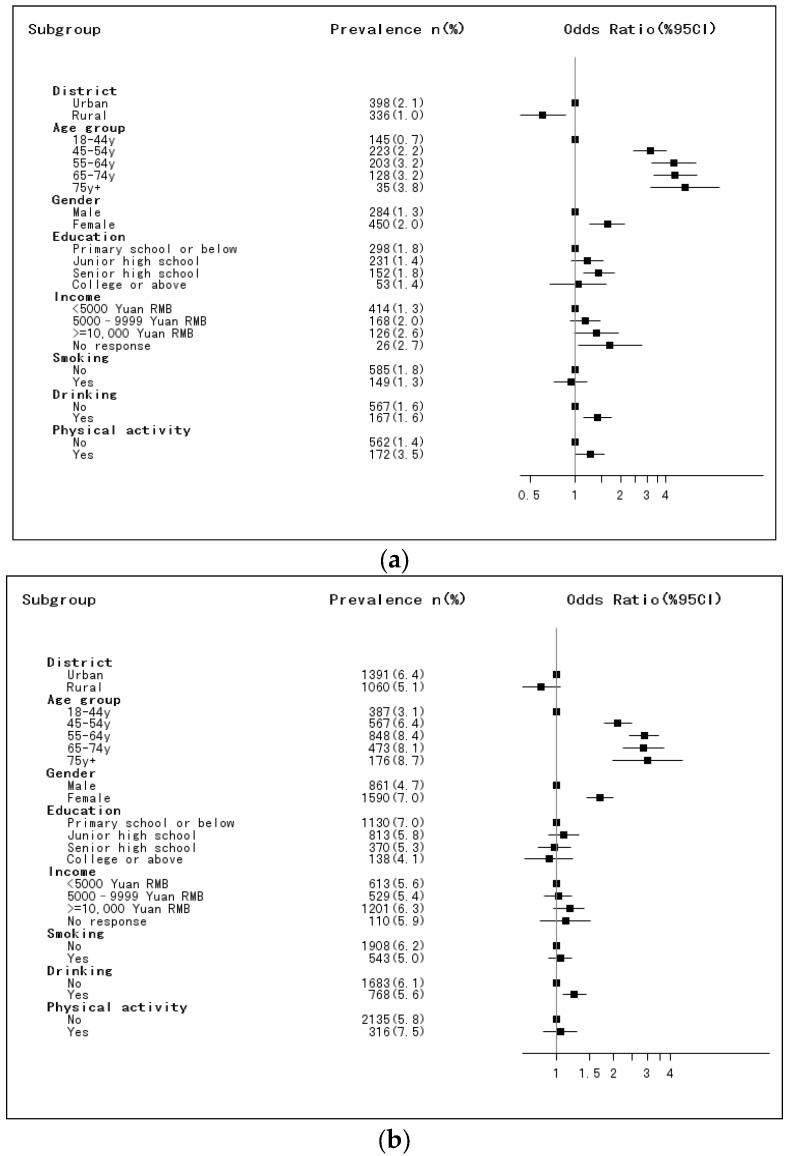
Association between socio-demographic factors or lifestyle and hypercholesterolemia in both surveys *; *: Models were adjusted for the variables in the figure. (**a**) Survey 2002; (**b**) Survey 2010–2012.

Older age, female, alcohol drinking was positively associated with hypercholesterolemia in both surveys (Figure 1). Residence, income, education, and physical activity were significantly associated with hypercholesterolemia in 2002 but not in 2010–2012.

There were no significant associations between hypercholesterolemia and rice, vegetables, fruits, eggs, milk, poultry, other red meat, cakes, sugar and salt intake both in survey 2002 and 2010–2012 after adjusting energy intake and socio-demographic and lifestyle factors (Table 5). Tuber intake was inversely associated with hypercholesterolemia in 2002 and 2010–2012 survey. Comparing extreme tertile of tuber consumption, the OR for hypercholesterolemia were 0.72 (95% CI: 0.55–0.94) in 2002 and 0.79 (95% CI: 0.69–0.91) in 2010–2012. There was an inverse association between fish consumption and hypercholesterolemia in both surveys. Compared with the first tertile of fish intake, the third tertile of fish intake was associated with a 50% increased likelihood of having hypercholesterolemia in both surveys. Wheat consumption was inversely associated with hypercholesterolemia in 2002 but not in 2010–2012. Pork and offal consumption were associated with increased likelihood of having hypercholesterolemia. Comparing extreme quartiles of pork consumption, the OR for hypercholesterolemia was 1.81 (95% CI: 1.45–2.24) in 2010–2012. In 2010–2012, hypercholesterolemia was inversely associated with other cereals intake (Q2 vs. Q1: OR = 0.83, 95% CI: 0.69–0.99, *p* = 0.035), legume intake (Q3 vs. Q1: OR = 0.67; 95% CI: 0.57–0.79, *p* < 0.001) and vegetable oil intake (Q4 vs. Q1: OR = 0.70; 95% CI: 0.58–0.83, *p* < 0.001).

Table 6 showed relationships between hypercholesterolemia and macro-nutrients intake. No association between carbohydrate and fat intake and hypercholesterolemia was found in both surveys, but protein intake was positively associated with hypercholesterolemia in survey 2002 (Q4 vs. Q1: OR = 1.96, 95% CI: 1.38–2.79, *p* = 0.003) and in survey 2010–2012 (Q4 vs. Q1: OR = 1.72, 95% CI: 1.24–2.40, *p* = 0.006). Moreover, protein from animal was positively associated with hypercholesterolemia in both surveys, the OR for hypercholesterolemia were (Q4 vs. Q1: OR = 2.04, 95% CI: 1.49–2.81, *p* < 0.001) in 2002 and (Q4 vs. Q1: OR = 2.04, 95% CI: 1.63–2.56, *p* < 0.001) in 2010–2012.While in 2010–2012, negatively association was found in protein from plant with hypercholesterolemia (Q4 vs. Q1: OR = 0.68, 95% CI: 0.54–0.87, *p* = 0.009).

## 4. Discussion

Based on the two national nutrition surveys, we found a four-fold increase in hypercholesterolemia prevalence among Chinese adults between 2002 and 2012.During the study period, there was a significant change in dietary intake especially a reduction the intake of carbohydrate. Women were more likely to have hypercholesterolemia than men in both surveys. Hypercholesterolemia increased with age. Protein and pork intake were positively associated with hypercholesterolemia. However, no consistent association between fruit and vegetable consumption and hypercholesterolemia was found in both surveys.

Unhealthy lifestyle factor such as smoking [12], alcohol drinking [13,14], sedentary lifestyle [15], overweight and obesity [16,17] are risk factors of hypercholesterolemia. Consistent with other studies, health-related behavioral factors, such as drinking was substantially associated with hypercholesterolemia in our study. The prevalence of alcohol drinking increased from 23.9% in 2002 to 33.6% in 2012. Due to the high prevalence of alcohol drinking, a high proportion of hypercholesterolemia can be attributed to drinking. Although smoking was less prevalent in 2012 as compared with 2002, the prevalence remained as high as 25.5%. Over the 10 years, the prevalence of overweight/obesity increased substantially (from 36.2% in 2002 to 45.0% in 2012). Overweight and obesity was a strong predictor for the incidence of hypercholesterolemia [18,19]. Consistent with other studies [20,21], we found that overweight (24.0 kg/cm^2^ ≤ BMI < 28.0 kg/cm^2^) and obesity (BMI ≥ 28.0 kg/cm^2^) was positively associated with hypercholesterolemia as compared with normal weight. The proportion of taking physical activity declined from 2002 to 2012 and physical activity seemed related to hypercholesterolemia, Since we do not have any information on the duration and intensity of the physical activity, it is difficult interpret the present findings. The increasing prevalence of hypercholesterolemia may be partly explained by the increase of the above unhealthy lifestyle factors.

The observed gender difference in total cholesterol levels in our study is consistent with other studies in China and some other developed countries [22,23,24,25]. However, an opposite direction of the association was found in Kuwait and Turkey, where men had higher cholesterol levels than women [26,27]. These inconsistent gender differences could be due to race, socioeconomic status, and dietary intake.

The association between socio-economic status and hypercholesterolemia varies by countries. Amiri et al. revealed that lower income was associated with a higher risk of hypercholesterolemia in Malaysia [28], while other studies have indicated no relationship between income and hypercholesterolemia [29].Educational level was related with the risk of hypercholesterolemia in a study in Korea [30]. However, in our study, higher income and higher education level had no relationship with hypercholesterolemia in both surveys. It may suggest that a high education level may not be necessary to have better nutritional knowledge or healthier behavior than those with a low education level. In fact, studies from China showing that people with high income were positively associated with a higher energy intake and risk of obesity [31].

The change in dietary intake is consistent with findings from the China Health and Nutrition Study (CHNS) [32]. Although the total energy intake decreased over the past 10 years, the distribution of macronutrient intake changed. The percentage of energy from fat increased significantly but percentage of energy from carbohydrate and protein decreased significantly from 2002 to 2012. These changes may be partly due to the availability of inexpensive vegetable oil and broader supply of animal foods especially pork over the study period [33].

In our study, the positive association between the intake of pork and protein and hypercholesterolemia is consistent with previous studies [34]. Pork and fish were main source of dietary protein and fat in our study and were positively associated with blood cholesterol level. A prospective study reported that high intakes of red meat were associated with increased risk, whereas intakes of nuts, fish and poultry were associated with substantially lower risk of developing coronary artery disease [34]. Compared with the Chinese dietary guideline on meat consumption, pork intake accounted for two thirds of the recommendation, which was almost more than 50 g in both surveys. Replace pork with lean red/white meat such as lamp/beef or poultry might be helpful to decrease serum total cholesterol concentration [35,36]. Protein from animal, but not plant sources, was associated with a higher prevalence of diabetes [37] as well as an increase of ischemic heart disease [38].In our analysis, animal protein was positively but plant protein was inversely associated with hypercholesterolemia. In general, plant-based foods (e.g., cereals, legume and tubers) were inversely related to hypercholesterolemia in our study. These foods are the main components of the Chinese traditional diet. Studies have shown that traditional Chinese dietary pattern is inversely associated with overweight/obesity [39]. Although rice intake has been shown to be associated with lipid profiles in Jiangsu Nutrition Study [40], we did not find a significant association between rice intake and hypercholesterolemia. However, the inverse association between wheat, tubers, legume as well as vegetable oil consumption and hypercholesterolemia in our study is consistent with previous findings [41,42,43].

The main limitation of the study is its cross-sectional study design. We cannot make conclusions on causation. Furthermore, the 3-day 24-h food intake may not be able to represent long-term dietary habits. Eating out is another source of potential measurement error. The strength of the study is that it is national representative sample and findings on the association between dietary factors and hypercholesterolemia can be generalized in Chinese adult population. The prevalence of hypercholesterolemia in our study should considered as age and gender standardized prevalence and the interpretation and comparison should be different from the previous findings in 2002 [5] and 2012 [6]. Furthermore, different from the prevalence study in 2002 by Zhao et al. [5], we used a different cutoff to define hypercholesterolemia (6.22 mmol/L [9] vs. 5.72 mmol/L [5]). Our study provided a detailed burden of hypercholesterolemia by socioeconomic and lifestyle factors. Findings from the study provide guidance for the prevention and intervention of dyslipidemia.

## 5. Conclusions

In conclusion, the study demonstrated a rapid increase in hypercholesterolemia prevalence between 2002 and 2012in Chinese adult population. Unhealthy lifestyle factors and change in traditional dietary pattern are positively associated hypercholesterolemia. There was a positive association between pork and protein intake and hypercholesterolemia. Intake of fruit and vegetable is not related to hypercholesterolemia in both surveys. Further research on the role of diet in the development and prevention of hypercholesterolemia is needed.

## Figures and Tables

**Table 1:** Demographic characteristics of participants between the two surveys.

	2002			2010–2012			*p* Value **
	Urban	Rural	Total	Urban	Rural	Total	
*N*	15,554	30,147	45,701	19,841	20,029	39,870	
Gender (*n*, %)							<0.001
Male	7150 (46.0)	14,205 (47.1)	21,355 (46.7)	8352 (42.1)	8970 (44.8)	17,322 (43.4)	
Female	8404 (54.0)	15,942 (52.9)	24,346 (53.3)	11,489 (57.9)	11,059 (55.2)	22,548 (56.6)	
Age (year, mean, se)	42.7 (0.4)	43.4 (0.3)	43.0 (0.3)	50.5 (0.7)	52.3 (0.6)	51.0 (0.5)	<0.001
Age group (*n*, %)							<0.001
18–44	6631 (42.6)	15,583 (51.7)	22,214 (48.6)	5547 (28.0)	6843 (34.2)	12,390 (31.1)	
45–54	3796 (24.4)	7403 (24.6)	11,199 (24.5)	4490 (22.6)	4785 (23.9)	9275 (23.3)	
55–64	2617 (16.8)	4341 (14.4)	6958 (15.2)	5293 (26.7)	4899 (24.5)	10,192 (25.6)	
65–74	2007 (12.9)	2268 (7.5)	4275 (9.4)	3310 (16.7)	2574 (12.9)	5884 (14.7)	
75–upper	503 (3.2)	552 (1.8)	1055 (2.3)	1201 (6.1)	928 (4.6)	2129 (5.3)	
Han population * (*n*, %)	14,726 (94.5)	26,421 (87.3)	41,147 (91.4)	18,875 (95.3)	17,057 (85.2)	35,932 (89.7)	0.669
Education level * (*n*, %)							0.248
Primary school or below	3848 (20.0)	15,272 (47.3)	19,120 (32.1)	5853 (27.4)	11,140 (56.3)	16,993 (36.6)	
Junior high school	5214 (32.5)	10,741 (37.4)	15,955 (34.5)	6878 (33.5)	6974 (33.6)	13,852 (33.5)	
Senior high school	4166 (29.8)	3395 (12.6)	7561 (22.2)	4449 (22.1)	1635 (8.2)	6084 (17.6)	
College or above	2326 (17.7)	739 (2.6)	3065 (11.1)	2661 (17.0)	280 (1.8)	2941 (12.2)	
Marital status * (*n*, %)							<0.001
Single	1281 (17.3)	1803 (11.3)	3084 (14.6)	932 (9.6)	726 (6.1)	1658 (8.5)	
Having a partner	13,232 (76.7)	26,677 (82.0)	39,909 (79.1)	17,200 (80.7)	17,935 (84.1)	35,135 (81.8)	
Divorced	230 (1.3)	208 (0.7)	438 (1.0)	349 (1.7)	164 (0.8)	513 (1.4)	
Widowed	811 (4.7)	1459 (6.0)	2270 (5.3)	1360 (8.0)	1204 (9.0)	2564 (8.3)	
Annual average income *^,^^&^ (*n*, %)							<0.001
<5000 Yuan RMB	6868 (45.1)	25,749 (85.6)	32,717 (63.0)	3034 (14.9)	7416 (38.5)	10,450 (22.4)	
5000–9999 Yuan RMB	4670 (29.6)	3069 (10.2)	7739 (21.0)	3665 (18.8)	5664 (27.6)	9329 (21.6)	
≥10,000 Yuan RMB	3462 (22.3)	922 (3.0)	4384 (13.8)	11,889 (59.5)	6388 (31.0)	18,277 (50.5)	
No response	454 (3.0)	407 (1.3)	861 (2.2)	1253 (6.8)	561 (2.9)	1814 (5.5)	

*: Adjusted by gender and age; ^&^: Annual average income per capital; **: *p* value was for the comparison between 2002 and 2010/2012 total.

**Table 2:** Lifestyle and serum total cholesterol concentrations of participants in 2002 and 2010–2012 surveys ^#^.

	2002			2010–2012			*p* Value **
	Urban	Rural	Total	Urban	Rural	Total	
TC * (mmol/L, mean, SE)	4.05 (0.05)	3.79 (0.03)	3.93 (0.03)	4.66 (0.03)	4.54 (0.04)	4.62 (0.02)	<0.001
Hypercholesterolemia (*n*, %)	398 (2.1)	336 (1.0)	734 (1.6)	1391 (6.4)	1060 (5.1)	2451 (6.0)	<0.001
Smoking ^##^ (*n*, %)							<0.001
Yes	3886 (26.3)	8519 (30.0)	12,405 (27.9)	4479 (23.1)	5734 (30.7)	10,213 (25.5)	
No	11,668 (73.7)	21,628 (70.0)	33,296 (72.1)	15,362 (76.9)	14,295 (69.3)	29,667 (74.5)	
Drinking ^&&^ (*n*, %)							<0.001
Yes	3636 (24.6)	6690 (23.0)	10326 (23.9)	6453 (33.7)	6506 (33.5)	12,959 (33.6)	
No	11,918 (75.4)	23,457 (77.0)	35,375 (76.1)	13,388 (66.3)	13,523 (66.5)	26,913 (66.4)	
Physical activity ^§^ (*n*, %)							0.195
Yes	3738 (20.0)	805 (2.9)	4543 (12.5)	3187 (14.1)	671 (3.3)	3858 (10.7)	
No	11,816 (80.0)	29,342 (97.1)	41,158 (87.5)	16,654 (85.9)	19,358 (96.7)	36,012 (89.3)	
BMI ^&^ (mean (SE), kg/m^2^)	23.6 (0.1)	22.4 (0.1)	23.1 (0.1)	24.0 (0.1)	23.2 (0.1)	23.8 (0.1)	<0.001
<18.5 (*n*, %)	770 (6.6)	2353 (9.0)	3123 (7.6)	704 (4.7)	1127 (7.2)	1831 (5.5)	<0.001
18.5–23.9 (*n*, %)	7480 (50.8)	18,689 (63.0)	26,169 (56.2)	9038 (47.0)	10,803 (54.7)	19,841 (49.5)	
24.0–27.9 (*n*, %)	5208 (30.7)	6925 (21.5)	12,133 (26.6)	7327 (34.6)	5973 (28.4)	13,300 (32.6)	
≥28.0 (*n*, %)	2096 (11.9)	2180 (6.6)	4276 (9.6)	2772 (13.6)	2126 (9.7)	4898 (12.4)	

^#^: Adjusted by gender and age; *: Total Cholesterol; ^##^: Smoking during the last 30 days; ^&&^: Drinking during the last year; ^§^: Exercising more than 150 min per week; ^&^: Body Mass Index; **: *p* value was for the comparison between 2002 and 2010/2012 total.

**Table 3:** Mean (SE) intake of food intake by gender and residence in two surveys *.

Food Items (g/Day)	2002			2010–2012			*p* Value **
	Urban	Rural	Total	Urban	Rural	Total	
Male							
*N*	7150	14,205	21,355	8352	8970	17,322	
Rice	223.5 (16.5)	260.8 (18.6)	240.5 (12.3)	125.8 (10.3)	236.3 (24.4)	163.9 (10.8)	<0.001
Wheat	136.1 (13.8)	193.7 (18.7)	162.3 (11.5)	140.4 (12.3)	161.9 (17.5)	146.8 (10.0)	<0.001
Other cereals	13.3 (2.0)	36.3 (5.8)	23.8 (2.9)	13.2 (1.8)	13.5 (2.1)	13.3 (1.4)	<0.001
Vegetables	262.0 (11.3)	321.6 (9.6)	289.2 (7.6)	285.0 (7.6)	257.1 (10.6)	275.2 (6.2)	<0.001
Fruits	59.6 (6.5)	29.2 (3.5)	45.7 (4.0)	44.3 (3.4)	27.5 (5.8)	38.0 (3.0)	<0.001
Eggs	34.4 (2.0)	20.6 (1.4)	28.1 (1.3)	28.8 (1.3)	17.8 (1.1)	24.8 (1.0)	<0.001
Fish	53.8 (6.5)	24.9 (3.1)	40.6 (3.9)	32.3 (3.2)	15.5 (2.4)	26.3 (2.2)	<0.001
Milk	54.4 (5.8)	8.8 (3.2)	33.6 (3.5)	35.3 (2.7)	7.6 (1.4)	25.3 (1.8)	<0.001
Poultry	24.5 (2.7)	10.7 (1.1)	18.2 (1.6)	16.1 (1.7)	12.6 (1.4)	14.8 (1.2)	<0.001
Pork	65.2 (3.7)	50.4 (3.2)	58.4 (2.5)	66.3 (3.2)	53.9 (4.3)	61.3 (2.6)	<0.001
Red meat offal	6.7 (0.7)	4.6 (0.5)	5.7 (0.5)	4.4 (0.4)	2.8 (0.5)	3.8 (0.3)	<0.001
Other red meat	16.7 (1.9)	8.3 (1.8)	12.9 (1.3)	14.1 (1.2)	6.7 (1.2)	11.4 (0.9)	0.001
Legume	16.7 (1.3)	18.4 (1.4)	17.5 (0.9)	16.5 (0.9)	12.7 (1.1)	15.0 (0.7)	<0.001
Tuber	28.7 (3.1)	64.3 (6.1)	44.9 (3.3)	34.5 (2.5)	49.3 (5.1)	39.4 (2.4)	<0.001
Nuts	5.6 (0.5)	3.6 (0.4)	4.7 (0.3)	5.1 (0.5)	2.7 (0.3)	4.2 (0.4)	0.002
Cakes	18.8 (1.7)	5.7 (1.2)	12.8 (1.1)	17.3 (1.3)	8.7 (1.6)	14.2 (1.0)	<0.001
Sugar	5.1 (0.6)	4.9 (0.7)	5.0 (0.4)	3.1 (0.2)	1.4 (0.2)	2.5 (0.2)	<0.001
Salt	10.3 (0.3)	14.1 (0.4)	12.0 (0.2)	9.2 (0.2)	11.7 (0.3)	10.1 (0.2)	<0.001
Vegetable oil	41.4 (1.2)	32.7 (1.8)	37.4 (1.0)	40.9 (1.4)	38.2 (1.8)	40.0 (1.1)	<0.001
Animal oil	2.7 (0.7)	11.5 (1.4)	6.7 (0.7)	1.6 (0.4)	7.1 (1.2)	3.5 (0.5)	<0.001
Female							
*N*	8404	15,942	24,346	11,489	11,059	22,548	
Rice	176 (12.1)	223.3 (14.7)	196.2 (9.4)	100.8 (7.4)	194.9 (18.7)	127.9 (7.5)	<0.001
Wheat	109.2 (10.5)	157.6 (14.1)	129.9 (8.6)	108.1 (8.7)	132.4 (13.6)	114.5 (7.3)	<0.001
Other cereals	13.7 (1.9)	32.3 (4.6)	21.6 (2.3)	13.3 (1.7)	14.0 (2.1)	13.4 (1.3)	<0.001
Vegetables	238.5 (10.7)	294.0 (9.0)	262.2 (7.3)	256.6 (7.2)	238.6 (9.7)	250.8 (5.8)	<0.001
Fruits	73.6 (7.5)	33.3 (3.9)	56.3 (4.7)	55.5 (3.4)	32.3 (4.8)	48.8 (2.8)	0.114
Eggs	31.2 (1.6)	18.4 (1.1)	25.7 (1.0)	25.5 (1.1)	16.5 (1.0)	22.8 (0.8)	<0.001
Fish	45.1 (5.9)	22.4 (2.9)	35.4 (3.6)	26.6 (2.9)	13.1 (2.2)	22.5 (2.1)	<0.001
Milk	57.7 (5.9)	8.1 (2.4)	36.5 (3.6)	39.0 (2.6)	7.0 (1.1)	29.6 (1.7)	0.009
Poultry	20.1 (2.3)	9.0 (0.9)	15.4 (1.4)	13.3 (1.5)	10.9 (1.3)	12.4 (1.1)	0.062
Pork	52.1 (3.2)	41.1 (2.7)	47.4 (2.2)	52.3 (3.0)	45.5 (3.6)	50.0 (2.4)	<0.001
Red meat offal	4.8 (0.6)	3.4 (0.4)	4.2 (0.4)	3.2 (0.3)	2.6 (0.4)	3.0 (0.2)	<0.001
Other red meat	11.8 (1.5)	6.4 (1.6)	9.5 (1.1)	9.6 (1.0)	5.3 (1.0)	8.3 (0.7)	0.001
Legume	14.1 (1.0)	15.8 (1.2)	14.8 (0.8)	14.5 (0.7)	11.2 (1.1)	13.5 (0.6)	<0.001
Tuber	27.0 (2.7)	59.8 (5.0)	41.0 (2.7)	36.3 (3.1)	47.5 (4.9)	39.5 (2.6)	<0.001
Nuts	4.8 (0.4)	3.1 (0.3)	4.0 (0.3)	4.6 (0.4)	2.5 (0.3)	4.0 (0.3)	0.442
Cakes	17.3 (1.4)	4.7 (0.8)	11.9 (0.9)	17.3 (1.0)	7.7 (1.4)	14.5 (0.8)	<0.001
Sugar	4.9 (0.5)	4.0 (0.5)	4.5 (0.4)	3.0 (0.2)	1.2 (0.1)	2.5 (0.2)	<0.001
Salt	8.6 (0.3)	12.2 (0.3)	10.2 (0.2)	7.7 (0.2)	9.6 (0.3)	8.2 (0.2)	<0.001
Vegetable oil	35.1 (0.9)	28.3 (1.6)	32.2 (0.8)	33.6 (1.0)	31.0 (1.4)	32.8 (0.8)	<0.001
Animal oil	2.3 (0.6)	10.2 (1.3)	5.6 (0.6)	1.6 (0.5)	6.2 (1.0)	2.9 (0.4)	<0.001

*: The figures were adjusted by age. **: *p* value was for the comparison between 2002 and 2010/2012 total.

**Table 4:** Energy, macronutrients, and percentage of energy in participants between the two surveys *.

	2002			2010–2012			p Value **
	Urban	Rural	Total	Urban	Rural	Total	
Male							
Energy intake (kcal/day)	2168.8 (45.5)	2628.5 (28.8)	2378.3 (28.2)	2042.2 (36.2)	2350.0 (58.8)	2150.0 (31.1)	<0.001
Carbohydrate Intake (g/day)	265.4 (7.9)	394.1 (4.7)	324.0 (5.0)	255.2 (6.4)	348.9 (10.6)	288.0 (5.6)	<0.001
Fat Intake (g/day)	87.2 (2.1)	79.6 (1.8)	83.7 (1.4)	85.2 (1.8)	79.2 (2.0)	83.1 (1.4)	<0.001
Protein intake (g/day)	71.7 (1.9)	74.0 (1.1)	72.7 (1.1)	66.2 (1.4)	63.7 (1.4)	65.4 (1.1)	<0.001
%E carbohydrate	50.0 (0.9)	61.6 (0.5)	55.3 (0.5)	50.1 (0.6)	59.2 (0.6)	53.2 (0.4)	<0.001
%E fat	36.3 (0.7)	26.9 (0.5)	32.0 (0.5)	36.9 (0.6)	29.9 (0.6)	34.4 (0.4)	<0.001
%E protein	13.6 (0.3)	11.5 (0.1)	12.7 (0.2)	13.0 (0.2)	10.9 (0.1)	12.3 (0.1)	<0.001
Female							
Energy intake (kcal/day)	1785.4 (30.0)	2207.4 (23.8)	1965.9 (20.2)	1666.9 (24.8)	1954.4 (44.2)	1750.8 (21.9)	<0.001
Carbohydrate Intake (g/day)	222.1 (5.6)	336.0 (4.0)	270.8 (3.7)	213.5 (4.4)	294.4 (8.1)	237.1 (4.0)	<0.001
Fat Intake (g/day)	72.9 (1.6)	67.7 (1.5)	70.7 (1.1)	70.0 (1.3)	66.2 (1.6)	68.9 (1.0)	<0.001
Protein intake (g/day)	59.7 (1.6)	63.0 (0.9)	61.1 (1.0)	54.6 (1.2)	54.4 (1.3)	54.6 (1.0)	<0.001
%E carbohydrate	50.2 (0.8)	61.7 (0.5)	55.1 (0.5)	50.6 (0.6)	59.4 (0.5)	53.2 (0.4)	<0.001
%E fat	36.2 (0.6)	26.8 (0.5)	32.2 (0.4)	36.5 (0.5)	29.6 (0.5)	34.5 (0.4)	<0.001
%E protein	13.6 (0.3)	11.5 (0.1)	12.7 (0.2)	12.9 (0.2)	11.0 (0.2)	12.4 (0.2)	<0.001

*: The figures were adjusted by gender and age; **: *p* value was for the comparison between 2002 and 2010/2012 total.

**Table 5:** Odds ratio (95% CI) for hypercholesterolemia by quartiles of individual food intake *.

	Q1	Q2	Q3	Q4	*p* Value
Survey 2002					
Rice	1	1.12 (0.85–1.46)	1.24 (0.92–1.68)	0.93 (0.65–1.33)	0.230
Wheat ^#^	1	0.97 (0.75–1.25)	0.58 (0.41–0.82)	-	<0.001
Other cereals ^&^	1	1.01 (0.85–1.20)	-	-	0.914
Vegetables	1	1.09 (0.81–1.48)	1.25 (1.01–1.56)	1.16 (0.83–1.64)	0.210
Fruits ^#^	1	1.40 (1.02–1.90)	1.15 (0.90–1.47)	-	0.097
Eggs	1	1.35 (0.39–4.70)	1.08 (0.87–1.34)	1.26 (0.98–1.62)	0.300
Fish ^#^	1	1.32 (0.99–1.76)	1.55 (1.21–2.00)	-	0.003
Milk ^&^	1	1.12 (0.87–1.44)	-	-	0.376
Poultry ^&^	1	1.19 (0.95–1.50)	-	-	0.129
Pork ^#^	1	1.11 (0.88–1.39)	1.28 (0.98–1.68)	-	0.193
Red meat offal ^&^	1	1.34 (1.00–1.80)	-	-	0.048
Other red meat ^&^	1	0.92 (0.69–1.24)	-	-	0.589
Legume ^#^	1	0.86 (0.66–1.12)	1.00 (0.80–1.25)	-	0.507
Tuber ^#^	1	0.76 (0.60–0.97)	0.72 (0.55–0.94)	-	0.025
Nuts ^&^	1	1.27 (1.00–1.62)	-	-	0.050
Cakes ^&^	1	1.00 (0.78–1.28)	-	-	0.982
Sugar ^&^	1	0.88 (0.70–1.11)	-	-	0.281
Salt	1	0.94 (0.71–1.24)	0.95 (0.73–1.24)	0.97 (0.76–1.24)	0.975
Vegetable oil	1	1.00 (0.74–1.34)	0.86 (0.65–1.13)	0.81 (0.61–1.07)	0.311
Animal oil ^#^	1	1.04 (0.72–1.52)	1.19 (0.90–1.58)	-	0.422
Survey 2010–2012	1				
Rice	1	1.05 (0.86–1.29)	1.22 (0.95–1.56)	1.25 (0.93–1.68)	0.377
Wheat ^#^	1	0.89 (0.76–1.06)	0.82 (0.66–1.02)	-	0.172
Other cereals ^&^	1	0.83 (0.69–0.99)	-	-	0.035
Vegetables	1	1.29 (0.91–1.53)	1.07 (0.89–1.28)	1.17 (0.94–1.45)	0. 202
Fruits ^#^	1	1.12 (0.91–1.38)	0.92 (0.81–1.05)	-	0.232
Eggs	1	0.95 (0.77–1.17)	1.10 (0.95–1.28)	0.96 (0.83–1.12)	0.357
Fish ^#^	1	1.11 (0.94–1.31)	1.51 (1.22–1.87)	-	<0.001
Milk ^&^	1	1.08 (0.93–1.26)	-	-	0.308
Poultry	1	1.19 (0.72–1.96)	1.22 (1.02–1.44)	-	0.074
Pork	1	1.10 (0.95–1.27)	1.29 (1.08–1.55)	1.81 (1.45–2.24)	<0.001
Red meat offal ^&^	1	0.99 (0.78–1.26)	-	-	0.945
Other red meat ^&^	1	0.96 (0.81–1.13)	-	-	0.625
Legume ^#^	1	0.87 (0.75–1.00)	0.67 (0.57–0.79)	-	<0.001
Tuber ^#^	1	0.89 (0.76–1.04)	0.79 (0.69–0.91)	-	0.004
Nuts ^&^	1	1.01 (0.84–1.21)	-	-	0.926
Cakes ^&^	1	0.90 (0.77–1.06)	-	-	0.223
Sugar ^&^	1	1.15 (0.96–1.37)	-	-	0.129
Salt	1	0.97 (0.81–1.17)	0.86 (0.72–1.03)	0.82 (0.67–1.00)	0.076
Vegetable oil	1	0.82 (0.69–0.98)	0.74 (0.60–0.90)	0.70 (0.58–0.83)	<0.001
Animal oil ^&^	1	1.25 (1.04–1.48)	-	-	0.014

*: Models adjusted for district, age, gender, nationality, education, marital status, income, smoking, drinking, physical activity, BMI and energy intake; ^#^: Food intake categorical levels were: none, below or above medium intake among participants; ^&^: Food intake categorical levels were: no consumption and consumers.

**Table 6:** Odds ratio (95% CI) for hypercholesterolemia by quartiles of individual macro-nutrients intake *.

	Q1	Q2	Q3	Q4	*p* Value
Survey 2002					
Energy intake	1	0.95 (0.75–1.21)	1.06 (0.81–1.38)	0.95 (0.70–1.30)	0.791
Carbohydrate	1	0.86 (0.66–1.11)	0.72 (0.51–1.02)	0.61 (0.39–0.96)	0.168
Fat	1	1.27 (0.94–1.71)	1.26 (0.95–1.67)	1.29 (0.90–1.85)	0.397
Protein	1	1.17 (0.94–1.45)	1.56 (1.17–2.08)	1.96 (1.38–2.79)	0.003
Protein from animal	1	1.08 (0.78–1.50)	1.66 (1.21–2.27)	2.04 (1.49–2.81)	<0.001
Protein from plant	1	0.98 (0.82–1.17)	0.72 (0.57–0.93)	0.84 (0.62–1.14)	0.048
Survey 2010–2012					
Energy intake	1	1.02 (0.87–1.19)	1.04 (0.88–1.23)	1.07 (0.90–1.28)	0.875
Carbohydrate	1	0.91 (0.76–1.09)	0.93 (0.76–1.15)	0.76 (0.56–1.03)	0.180
Fat	1	1.15 (0.93–1.41)	1.05 (0.86–1.30)	1.12 (0.87–1.44)	0.583
Protein	1	1.30 (1.09–1.57)	1.35 (1.10–1.66)	1.72 (1.24–2.40)	0.006
Protein from animal	1	1.22 (1.04–1.43)	1.59 (1.30–1.94)	2.04 (1.63–2.56)	<0.001
Protein from plant	1	0.88 (0.75–1.02)	0.78 (0.65–0.94)	0.68 (0.54–0.87)	0.009

*: Models adjusted for district, age, gender, nationality, education, marital status, income, smoking, drinking, physical activity, BMI and energy intake (except when energy intake was the exposure variable).
